# Cash efficiency for bank branches

**DOI:** 10.1186/2193-1801-2-334

**Published:** 2013-07-22

**Authors:** Julia García Cabello

**Affiliations:** Departamento de Matemática Aplicada, Facultad de Ciencias Económicas y Empresariales, Campus de Cartuja, Granada, 18071 Spain

**Keywords:** Stochastic model, Function of cash flow costs, Cash optimization, Transaction demand for cash, Branches

## Abstract

Bank liquidity management has become a major issue during the financial crisis as liquidity shortages have intensified and have put pressure on banks to diversity and improve their liquidity sources.

While a significant strand of the literature concentrates on wholesale liquidity generation and on the alternative to deposit funding, the management of an inventory of cash holdings within the banks’ branches is also a relevant issue as any significant improvement in cash management at the bank distribution channels may have a positive effect in reducing liquidity tensions. In this paper, we propose a simple programme of cash efficiency for the banks’ branches, very easy to implement, which conform to a set of instructions to be imposed from the bank to their branches. This model proves to significantly reduce cash holdings at branches thereby providing efficiency improvements in liquidity management.

The methodology we propose is based on the definition of some stochastic processes combined with renewal processes, which capture the random elements of the cash flow, before applying suitable optimization programmes to all the costs involved in cash movements. The classical issue of the Transaction Demand for the Cash and some aspects of Inventory Theory are also present.

**Mathematics Subject Classification (2000)**

C02, C60, E50

## Introduction and links with the related literature

Along with risk, liquidity management represents the main rationale for the existence of banks in the classical financial intermediation theory (see, for example, (Allen F and Santomero AM 1998) or (Allen F and Gale D 2004). In the standard framework, the basic management challenge as far as liquidity is concerned is how to cover depositors’ random consumption needs and how to set the subsequent deposit insurance mechanisms for these depositors, (Diamond DW and Dybvig P 1983).

During the financial crisis that started in 2007, bank liquidity has become a major issue in a context of uncertainty and instability. Liquidity tensions have made banks develop several strategies to retain depositors and to manage liquidity as efficiently as possible to avoid financial fragility that, overall, makes the whole financial system weaker (Diamond DW and Rajan RC 2011).

Several studies have dealt with the implications of wholesale debt markets of retail deposit markets on liquidity tensions as well as on the possible policy actions to solve such market-related liquidity shortages (as shown, inter alia, by (Fecht F et al. 2011) or (Loutskina E 2011). Since the notion of liquidity management comprises all short- and medium-term cash flows no matter whether they are accounting-based or cash, in this paper we focus on possible improvements in optimization of cash inventories within banks and, in particular, on how to improve cash management inventories at branch level, as the scope of this paper deals essentially with the cash part in ATMťs and for the cash desks.

Although there are significant potential efficiency improvements in liquidity from branch-level cash management, there are only a few studies dealing with these issues, as most of the banking models in the financial literature assume the standard allocation models of cash from central hubs to branches as given. A relevant exception is the work of Pokutta and Schmaltz (Pokutta S and Schmaltz C 2011) who study how to improve the optimal allocation of cash at large banking groups. They compare the alternatives of a central liquidity hub and the case of many decentralized branches. They provide an analytical solution for a 2-branch model and show that a liquidity center can be interpreted as an option on immediate liquidity where the value can be interpreted as the price of information. Importantly, Pokutta and Schmaltz derive the threshold above which it is advantageous to open a liquidity center and show that it is a function of the volatility and the characteristic of the banking network.

Other studies have also dealt with liquidity management not just at the bank level but as a general feature of firm management. In this front, some papers have made use of stochastic and inventory theory to propose some models of firms’ cash management. Among these approaches, the closest to our empirical aims is Ferstl and Weissensteiner (Ferstl R and Weissensteiner A 2008). They consider a cash management problem where a company with a given financial endowment and given future cash flows minimizes the Conditional Value at Risk of final wealth using a lower bound for the expected terminal wealth. Ferstl and Weissensteiner use a multi-stage stochastic linear program (SLP) where a company can choose between a riskless asset (cash), several default- and option-free bonds, and an equity investment, and rebalances the portfolio at every stage. They explicitly estimate a function for the market price of risk and change the underlying probability measure and simulate scenarios for equity returns with moment-matching by an extension of the interest rate scenario tree.

Our paper uses optimization theory as well as stochastic calculus to develop a model of managing an inventory of bank cash holdings that provides benefits as compared to the standard branch cash management models. Although the introduction of Automatic Teller Machines and other technological innovations has reduced cash management costs, there is still a pressing need to optimize resources, imposed by the high competition among bank institutions in the present scenario of economic and financial crisis. In this sense, this paper makes a proposal for the optimization of cash at branch level. In the banking world, several companies offer such services of optimization to banks and retails companies. These solutions are based on Information Technology (IT) which built internal cash cycles as a special form of supply chain: cash should be either made available at the right time in the right quantity or to be returned to the cash cycle. However, although the solutions offered by this kind of companies appear as effective in minimizing costs associated with cash flows, they are too expensive to be implemented in branches. The reality is that all branches (small, medium or big sized) *do not use* such kind of IT methods to optimize their cash, which probably remain reserved to the management of big cash centers of the bank companies (if so).

Thus, this paper represents a concrete programme of optimization of the bank cash for all branches. This program consists of some precise, simple formulae which could be implemented in branches at a set of instructions from the bank company. Among the major virtues of the program that we propose are that these formulae are *very easy* to implement and *practically cost-free*.

Specifically, we propose to substitute the old procedures the branches use (and abuse), based on their historical data, by mathematical formulae. These old methods use previous data in similar circumstances (similar day, similar month, working day or saint day) as a process of trial and error for the present day, being this one the unique reference in the daily decision-making on cash flow. Moreover, this exercise of historical data implicitly carries a human error of valuation, since most of the process is based on the valuation of the person in charge of the branch, who decides, after consulting the previous information, which quantity it must demand for (if equal, higher o lower to that of the historical data). On the contrary, the mathematical tools that we have used, which combine optimization programmes with stochastic calculus as well as some aspects of Inventory Theory, conform a rigorous and powerful weapon to reduce cash inventory as well as to optimize the use of fixed assets.

This problem can be related with a classic issue: the transaction demand for the cash, which began with Baumol (Baumol WJ 1952) and Tobin (Tobin J 1956), and more recently with Álvarez and Lippi (Álvarez F, Lippi F 2009). The transaction demand for the cash consists of managing an inventory of cash holdings: the decision maker holds two distinct types of assets, one asset which bears interest at a given rate, and a noninterest bearing asset where periodic receipts of deposit and expenditures are made. Transfers of funds between the two accounts are permissible but at a cost (transfer cost).

There are also other costs of different nature involved: the opportunity costs derived from the fact that funds into the noninterest bearing asset are losing money while they are not into the interest bearing portfolio. Apart from the opportunity costs associated with cash inventories, there are many others: personnel costs, investments costs, material and clearing costs, insurance and logistic costs for transport and handling, (Miller M and Orr D 1966).

The structure of this paper is as follows: section “The mathematical model” is devoted to describing in mathematical terms the functioning of the cash flow of any branch, by separating the different types of deposits and expenditures and modeling them. In this section, we also choose the variables and we design a proper objective function which captures all the costs involved in the cash movements. In section “The random deposits and expenditures”, we catch the random elements of the model, such as number of branch users or number and quantities of cash movements (deposits and withdrawals), by defining suitable Poisson (compound) processes. In addition, we extract some useful properties to be used in later sections.

Section “Instructions from the bankťs company to its branches” contains the precise instructions that the bank company must impose to its branches in order to optimize its liquid capital resources. These instructions are given in the shape of mathematical formulae to calculate the required amounts of cash and they substitute the old methods based on historical data. In addition, in this section we also examine the standard deviation as the measure of the committed mistake in the approximation process, to make a first approach on deciding for which kind of branches work more effectively the proposals made in this paper.

In section “The underlying optimization problem”, we deeply analyze and resolve the key optimization problem of this paper as we determine the required amounts of cash of any branch, taking into account the particular features (needs) of any of them.

Section “Comparative statics” is devoted to comparative statics examples on some parameters of the model, to help understand under which conditions these efficiency gains will be larger or smaller. Finally, in section “Conclusions”, the set of all found conclusions in section “Comparative statics” is summarized and interpreted into economical terms, depending on the values of the different estimators (parameters) that we previously defined.

## The mathematical model

Our intention is to construct a mathematical model that comprises the whole economic setting of cash moves (in the scenario of the bank branches).

Different approaches to the problem can be made in order to optimize the bank cash handling: in the first place, a global approach, modeling the situation of a bankťs company viewed a *solid* piece of the puzzle. In this case, we have to consider the set of international rules as constrains to the optimization program. This course of action is very complicated because the cash movements are not on the surface. In fact, the cash funds are strongly tied up as they are invested in many other bank products.

As for the present paper, we have chosen to consider any bankťs company as a institution *composed by several pieces* in contrast with the unique and solid piece of the global point of view. The pieces which conform a bankťs company are its *branches*: hence, we will carry out the optimization process across all the branches.

Then, let us start with a few words on the functioning of the branches. In their labor of attention to the users, any branch must have a quantity of ready money, which comes in part from the cash central, as well as from the deposits made by the individual users or companies.

Periodically, the branch adjusts its cash to its necessities -deposits and expenses- avoiding generating a quantity of dormant money. After this adjustment, both of the following possibilities may take place: the branch has generated a surplus of cash or, on the contrary, the branch needs an injection of money.

In both cases, the branch needs help from its cash central (the closest one): in the first case, the branch requires that an armoured van evacuates the surplus of money. In the second one, that of the branch needing more cash, the cash central should move money to the branch, by means of an armoured van as well. In any case, these cash movements from the cash central to the branches take place after a *concrete demand of money from branches to the cash central*. Later on, we specify how it works in the real world.

As for the demand of money from branches to the central office, this often occurs with a *forecast of funds according to a concrete demand*. This is the case, for instance, of users who want to cancel a deposit and to recover their money. Hence, *the branch can anticipate this expenditure*, in time and in quantity. This argument of anticipation of the bank in some cases is based on the real normative of some bank companies, who require that clients announce the physical withdrawal one day before such that the bank has twelve hours to demand the funds from its headquarter.

The above example suggests that there are two types of expenditures: those which can be anticipated and those of a strictly random nature. The same classification can be established in the case of deposits. Thus, we refer to both the above types as *expected and unexpected expenditures and deposits*.

Turning again into the movement of money from the cash central, this event implicitly carries a cost that we analyze deeply in later sections. Mainly, it consists of a *logistic cost for transport and handling*, due to the costs generated by renting a security company (which includes costs for security personnel and costs for transport in armoured vans). There is involved as well an *opportunity cost*, derived from the fact that funds into the non interest bearing asset are loosing money while they are not in the interest bearing portfolio. One of the targets of this paper is, then, to minimize the different costs implicated in the currency management while keeping the customer satisfaction as high as usual.

### Dynamics of the funds of a branch

In order to build a realistic (mathematical) model, let us recall the movements of the cash flow of the branches.

For simplicity, we will assume that the bank capital can be separated into two products: cash funds and the rest of bank goods, even those with high capacity of turning out to be in ready money without loss of their value. This hypothesis can seem naive, but it allows us to be focused only in the liquid assets of the bank company.

As we mentioned before, each branch of any bank company has to assume several periodic expenses, some of them are predictable while many others are not. This implies that an entry of money must exist, available when necessary, from the closest cash central. This channel of entry of money is complemented with the daily deposits that the individual users or companies make.

The cash central sends cash to the branch *after* a concrete request from the branch. This request is done after adjusting both the programmed expenses and those which are of random nature, with the own expected deposits and the cash available in the branch at this moment. All this process is done with the target of not generating a surplus of money.

In the real world, the criterion with which such a request is done is purely based on the *historical data* of the branch. This expresses that the branch compares such a day as today with a similar day of the past. The concept of “similar day” means a day of chosen similar characteristics: working day versus saint day, day at the beginning of the week versus day at the weekend, etc. The branch, having identified such a similar day, examines the success or failure of the quantity required for such a day of the past and does its request after valuating if doing so in an equal, higher or lower quantity.

As it is the banks company who makes this optimization process, it establishes some mechanism of control on the branches to restrict these movements of entries and exits of money.

1. A first mechanism of control to optimize its resources is set by the bankťs company by fixing a certain number of periodic *stops*: branches call *stop* to each of the armoured van stops next to the branch. Each branch has a certain number of stops available which might or might not be required. Let us recall that stops occur after a request of the branch, and due to both possibilities: caused by the existence of a surplus of cash or, on the contrary, caused by the absence of cash to cover all the branches necessities.
2. The branch will know that there is not enough cash when they will not be able to cover all the programmed (or unexpected) expenses. However, the existence of a surplus of ready money can be established *only with a comparative to a reference*, which allows us to introduce the second mechanism of control on behalf of the bankťs company.

Such reference to set how much money exceeds *in this branch* consists of an upper bound *C*_*z*_ (maximum of cash), which is fixed by the bankťs company for each branch, attending some parameters, particularly, the volume of the branch turnover which, as we will see later, can be identified with the size of the branch. In comparison with *C*_*z*_, if the branch liquid funds exceeds this margin or not, the branch will know if it must require or not one stop to evacuate the surplus.

Although we will return to this point later, let us observe now that in addition, and depending on the internal normative of each bank company, the bank can decide (or not) to add a bit more over *C*_*z*_ for precautionary motives (i.e., for prevention) in order to fulfill the clients demand up to a certain confidence level. Since such precautionary “bit more” will be fixed for each bank company in attendance of their internal rules of functioning, we calculate along this paper the optimum *C*_*z*_ for minimizing the bank costs assuming that this precautionay “bit more” is external to *C*_*z*_ and hence, it is not included in *C*_*z*_. Then, once we have finished our task of determining the optimal amount *C*_*z*_ for minimizing bank costs for *any* bank company, each bank could (or not) add more o less precautionary amount, following its internal normative.

### Development of the model: election of variables and function of costs

The main purpose of this paper is to optimize the bank cash, as we said before, across the optimization of the funds of any of its branches. The final objective of this optimization process is, thinking as if we were the bankťs company, to provide to the branches with some precise instructions under the shape of formulae, which will be deduced in later sections. These instructions will constitute a set of simple formulae, with no cost in their implementation in practice. These features conform the more valuable advantages of our approach compared with other techniques for different bank branches. In short, the method that we propose is

-  easy to implement, what means among other things that there is no need of additional formation for the branch staff to manage this, which would represent an extra cost and
-  almost cost-free, since it can be implemented simply as a set of new instructions from the bank to their branches.

Let us start by choosing the variables of our model: the first one is the *total amount of money that the branch demands from its cash central*, *C*_0_. The requirements for *C*_0_ are that it should

-  be enough to cover both expected and unexpected expenditures of the branch and
-  minimize the costs function of the bankťs company.

A formula to calculate an accurate *C*_0_ for each monetary circumstances of each branch, will be deduced later on. Let us note that a negative *C*_0_ should mean that there is a surplus of cash in the branch.

From the bankťs company point of view, the second variable to be considered is the upper bound (the maximum) of cash resources that the bankťs company fixes for each branch as a reference to determine if a surplus of cash exists in there. Let *C*_*z*_ be the maximum point of the cash level which the bankťs company will allow in its branches. This variable depends on some other endogenous variables, such as the geographic location or size of the branch. However, for simplicity, we reject these features of *C*_*z*_ to center our attention on the main requisite on *C*_*z*_ of minimizing costs to the bankťs company.

Thus, we assume that the cash balance in the bank branch is allowed to fluctuate until it reaches the upper bound, *C*_*z*_. Once the branch exceeds the quantity *C*_*z*_, it must demand from the cash central one stop to evacuate the surplus of money.

After having selected the variables, let us continue with the banks objective function. For this, we follow the standard practice in inventory theory by assuming that the bank seeks to minimize the long-run average cost of managing the cash balance under some *policy of simple form*. On this basic function, we will add later some modifications, specific to our particular case.

On the two variables, *C*_0_ and *C*_*z*_, we will make use of the following bankťs objective function *ε*(*C*_0_,*C*_*z*_) as starting point to construct the final function of costs. *ε*(*C*_0_,*C*_*z*_) is defined as

(See (Baumol WJ 1952; Miller M and Orr D 1966; Tobin J 1956) and (Álvarez F, Lippi F 2009) for references on *transactions demand for cash*). In a parallel form to the classical situations in transactions demand for cash, we have in our particular context of bank branches two-asset setting, one asset being the branchťs cash balance, and the other, a separated portfolio of liquid assets, represented by the cash central.

We derive the above function in a similar way to (MillerMand Orr D 1966). Also, the main assumption of the model will be established here: in contrast with the (deterministic) Baumol model, now the net cash flow is completely stochastic. Mainly, the branch cash balance will be allowed to fluctuate freely until it reaches either the lower bound, zero, or the upper bound, *C*_*z*_, at which time a transfer of founds will be undertaken to restore the balance to a security level according to the normative. By the moment, we consider two separated addends, one for the costs derived from the cash flow and the other for the *opportunity costs* associated with cash inventories, that is, the costs that the bankťs company suffers for not having the cash in other products that yield higher benefits.

As for the first addend, this can be written as the product of the cost per transfer (say *γ*) and the total expected number of transfers per period of time considered. While in the classical issue of *transactions demand for cash*, the costs due to cash flow are simply *transfer costs* (from one current account to another), in our particular context, these costs include personnel costs, investments costs, material and clearing costs, insurance and logistic costs for transport and handling.

Hence, this first addend of the function is

where *A* denotes the variance of daily changes in the cash balance. Specifically, considering that the random behavior of the cash balance can be characterized as a sequence of independent Bernoulli trials, if *μ* denotes the amount of euros that the branch cash balance increases or decreases in some small fraction of a working day , thus *A* = *μ*^2^*t*.

As for the the costs per stop, *γ*, we simply distributes the total costs per contracting a security company (which are constant and in consequence, independent of the amount transferred) by the mean of total stops for this branch. Let us point out that in reality, branches (bank companies) pay a *constant* amount per month for all services to the security company, independently of the number of stops they have to make.

As for the second addend, this will be the product of the daily rate of interest earned on portfolio (e.g., other banks products which yield higher benefits), say *ν*, and the average daily cash balance. This steady-state distribution of cash holdings is of a discrete triangular form with base *C*_*z*_ and mode *C*_0_. Hence the mean of such a distribution is .

Then, summarizing all the above information, the objective function till now is

where *γ* represents the costs per stop,  is the total number of stops in the period of time, while *ν* is the daily rate of interest earned on the portfolio, and  represents the average daily cash balance on bank branch.

However, we must also consider other costs involved with a large quantity of *stored* money inside its branches: the payment on the bankťs company to a theft insurance policy. This cost will be directly proportional to the maximum amount of money allowed by the bankťs company for this branch in particular.

Hence, the completed objective function is

Note that in the objective function (in the first addend) there is a parameter directly related to the branch users behavior. This is *μ*, with *A* = *μ*^2^*t*, which acts as an index of how active the branch users are: the more times they make withdrawals or deposits -and the higher are the quantities they move- the bigger is *μ* and the bigger are the fluctuations of the cash balance. Hence, from now on, we consider *μ* as the **indicator of the fluctuations of the branch cash flow**. Let us remark that *μ* ∈ [0,+*∞*) if it is considered as a symmetric function:

In practice, *μ* is strongly directly related with the *geographic location* of the branch, more than to its size: nearer the branch is situated to an industrial, commercial or financial center, higher will be the fluctuations of the branch cash flow, so higher will be *μ*.

Later on, in section “Comparative statics”, we will define a second parameter which we will use to calibrate the strength of our model. This will be the proper branch size: both parameters will allow us to measure the changes of the optimal policy for branch costs, depending on the different values on these estimators.

## The random deposits and expenditures

As mentioned before, the quantity *C*_0_ of money that the branch demands to its cash central must fulfill some requirements, which can be condensed in only one: *C*_0_ must cover the expenses of the branch during the considered period of time.

Following section “The mathematical model”, the branch expenses can be classified into *expected and unexpected* expenditures. While the first ones are easily modeled like a constant (which could be left aside of the model, as we will argue in short), the second ones are much more difficult to be designed, due to their random nature. This section is, thus, devoted to capturing the randomness of our problem by means of some stochastic processes.

Through the analysis of our problem, some stochastic elements have been kept hidden: first of all, the number of bank branch users during the considered period of time. These consumers make deposits and withdrawals through the available ATM of the branch as well as from the cash desks. A second stochastic element involved is the quantities of money that these branch users take into and out. Note that the bank branch deals with both stochastic elements using its historical data.

Let *N* be the number of costumers which make use of the bank branch during the considered period of time. Let us recall that often, the arrival process of customers can be described by a *Poisson process*. Mathematically the process is described by the so called *counting process* *N*(*t*) or *N*_*t*_. The counter tells the number of arrivals that have occurred in the interval (0,*t*), that is,

From this definition and considering the period of time as the unit of time, note that *N* = *N*_1_.

For that reason, if *N*_*t*_ is a (Poisson) counter process of parameter, say, *λ*, some of its properties are the following:

-  the number of arrivals to the bank branch in an interval of length t has a Poisson distribution with parameter *λ*·*t*; that is,  measures the probability of *n* bank branch customers in the time *t*.
-  The mean and variance of *N*_*t*_ are

Particularly, since *λ* = *E*[*N*], follows that the rate of the Poisson process *N*, *λ*, is the average of branch consumers in the considered period of time.

In order to properly structure the later withdrawals and deposits movements, note now that not all the users who come to a branch do so in order to make withdrawals or deposits. For this reason, the counting process *N*_*t*_ can be separated into many counting processes, which we summarize for simplicity like

where  represents the number of withdrawals in the interval (0,*t*),  represents the number of deposits made in the interval (0,*t*) and the term *O*_*t*_ gathers the rest of operations and requests (apart from withdrawals and deposits) made by the branch users.

Apart from the number of branch consumers per period of time, another random element of our problem is the amount of each withdrawal made by each consumer. Let *W*_*i*_ the withdrawal that the *i*-th user makes. We capture all these quantities *W*_*i*_ by means of a compound Poisson process.

A compound Poisson process is a (random) stochastic process with jumps. The jumps arrive randomly according to a Poisson process and the size of the jumps is also random, with a specified probability distribution. In our context we define *the withdrawal process*, parameterized by certain rate *λ*, as the compound Poisson process given by

viewed as independent and identically distributed (i.i.d.) random variables. Then, *X*_*t*_ is the total amount that has been taken off by the  bank branch users until the moment *t* of the considered period of time. Note that, particularly,

is the total amount of money that is taken from the bank accounts -either through the ATMs or through the cash desks- for *t* = 1.

At this point, remember that the mean of a compound Poisson process can be calculated via the mean of one of the i.i.d. variables, as

where *λ*^*w*^ is the rate of the Poisson process .

Consequently, for *t* = 1, we have

Once we have modeled the withdrawal process, we can operate in a similar manner for the deposit process. That is, the *deposit process* parameterized by certain rate *λ*^*d*^, as the compound Poisson process given by

where *D*_*i*_ denotes the deposit that has been made by the *i*th-customer, viewed as independent and identically distributed (i.i.d.) random variables. Then,

In a parallel form to the withdrawal process, the mean of this compound Poisson process can be expressed

where *λ*^*d*^ is the rate of the second Poisson process .

For *t* = 1, we also have that

We will complete this section by calculating the number of withdrawals and deposits in terms of expected values of expected times between two arrivals as well. This will be done using some classical results cited in (Álvarez F, Lippi F 2009).

For this, let us remember first some previous definitions and constructions.

In a counting process, often *T*_*n*_ represents the time between consecutive arrivals: specifically, *T*_*n*_ denotes the time between the (*n* − 1) − *th* arrivals and the *n*-th one. These variables are i.i.d., and they allow the constructions of a new variables,

which represent the time until the *n*-th arrival.

By defining *N*(*t*) = *N*_*t*_ := *max* {*n*/*τ*_*n*_ ≤ *t*}, this counts the number of arrivals which occurs in the interval (0,*t*), in the same manner that, in our particular context, *N*_*t*_ counts the number of bank branch users in the interval (0,*t*), as we mentioned before.

Hence, the *renewal function* is defined by

If we denote by *μ* the expected time between arrivals, the fundamental theorem of Renewal Theory states that

This means that the expected number of arrivals is equal to the reciprocal of time between arrivals. As a logical consequence of this result, minor all is the number of users who extract money, major will be the time that passes between the two arrivals.

This result can be applied when the arrivals are withdrawals and deposits, respectively. For this, let us define new random variables which will measure the times between withdrawals or deposits.

Let

be the time until the *n*-th arrival of an user who makes an withdrawal.

By defining , this counts the number of withdrawals which occur in the interval (0,*t*). In the same line, the *renewal function* for withdrawals is defined by

As direct application of the fundamental theorem of Renewal Theory, the expected number of withdrawals is equal to the reciprocal of time between withdrawals.

On a parallel form, this process can be defined for deposits: let

represent the time until the *n*-th deposit has been made.

By stating , this counts the number of deposits made in the interval (0,*t*). Therefore, the *renewal function* for deposits is defined by

which expresses, by means of the fundamental theorem of Renewal Theory, that the expected number of deposits is equal to the reciprocal of time between deposits.

In both cases, the number of withdrawals and deposits -the first term in the equality set by the fundamental theorem of Renewal Theory- is given by *N*^*w*^ and *N*^*d*^ respectively, considering the period of time as the unit.

As for the second term in the fundamental theorem of Renewal Theory, the reciprocal of time between two withdrawals or deposits,  and , let us examine it immediately afterwards.

We start with *μ*^*w*^, the time between two withdrawals. The Poisson (counting) process  with intensity  (particularly *N*^*w*^ with intensity *λ*^*w*^) is also an renewal process where the time between two withdrawals, , is distributed as an exponential with parameter *λ*^*w*^. So simple algebra shows

The same idea works for deposits. Thus, *μ*^*d*^ denotes the time between two deposits,

Therefore, the number of withdrawals and deposits per unit of time is given by the following formulae:

as well as

## Instructions from the bankťs company to its branches

As we mentioned before, the central aim of this paper is to optimize the bank cash inventories across its branches. For this, our proposal is that the bankťs company must *impose some restrictions* to its branches cash movements. These restrictions are imposed in the shape of some formulae which help the branches to calculate their needs of cash, before they make a cash requirement to the cash central. These formulae accurately adjust the demand for money to the real needs of the branches, avoiding a surplus of money and, hence, minimizing the opportunity costs.

This section is devoted to rewriting these restrictions, designed as formulae, which the bankťs company imposes to its branches as the *new* mechanism to calculate the required cash. In fact, these formulae would substitute the old mechanism the branches use until now, based only on their historical data. Actually, since all work has been done in the previous sections, we are now ready to extract conclusions.

The branches cash needs, *C*_0_, are quite simple to define in view of the deposits and withdrawals of money: they are equal to the total amount of expenditures minus the total amount of deposits (expected and unexpected). Let us associate the expected quantities of money as well as the unexpected ones in separated addends, since both addends exhibit opposite qualities. Thus,

assuming that both expected expenses and expected deposits are constant since they could be anticipated because they do not exhibit any random features. Let *K* be this constant which brings together both expected expenses and deposits.

As the expected moves of cash, represented by the constant *K*, do not exhibit unpredictable behaviors, it is logical to assume that they could be left aside of the model. That means that, from now on, we will be focused *only on unexpected* moves of cash (withdrawals and deposits) with the concrete intention of finding a formula which, as easy as possible, approximate them. Once we have concluded our task, the expected moves of cash *K* will be simply added to our formulae.

Another reason for leaving aside of the model the quantity *K* derived from expected movements of money, is that this may be considered as part of the *security cash level* (like settlement accounts) that each bank holds in order to fulfill the clients demand up to a certain confidence level for precautionary motives. As we mentioned in previous sections, the total amount of cash which the bank holds for security reasons (*K* is part of this) depends mainly on the internal normative (or rules of functioning) of each bank.

Let us return then to the task of estimating the unexpected cash moves of the branch, *X*_1_ and *Y*_1_. If we approximate each random variable *X*_1_ and *Y*_1_ by its mean, we have that

Then, using the specifications made for *E*[*X*_1_] and *E*[*Y*_1_] in the former section, the above formula turns out to be

Thus, this formula is the new mechanism of control that the bankťs company must impose in its branches in substitution of their old methods, based on historical data. The introduction of this formula must be imposed as part of the program of minimizing costs for internal cash flow. In the light of this, we emphasize that this formula is very simple itself as well as very simple to manage with.

Let us make now an slight approach to the adequacy of the model for different branch sizes, by evaluating the error committed in the approximation used before on the random variables *X*_1_ and *Y*_1_.

The previous formula is constructed by means of the stochastic variables, *X*_*t*_ and *Y*_*t*_ (the so called withdrawal process and the deposit process, respectively) which captures both random processes of withdrawing and incoming money, for a random number of users, *N*(*t*). Moreover, in order to transform into determinist a random problem as far as possible, we have used the approximation of the stochastic variables *X*_*t*_ and *Y*_*t*_ by their means:

As any approximation, this carries a little error of calculus. We are now going to examine the committed mistake of approximation as well as to interpret this for different branch sizes.

For any random variable, the mentioned miscalculation is given by its *standard deviation*. As it is well known, the standard deviation of a random variable *X* is defined as the quadratic root of its variance and it provides a measure of the dispersion of the variable: minor is the standard deviation, major will be the concentration of data about the mean.

Let us recall that, from the definition of variance of any compound Poisson process, the variance of the random variables *X*_*t*_ and *Y*_*t*_ are

Henceforth, in the particular case of *t* = 1, theses variances are

or, even more,

Then, in our particular context, the measure of this error is given by

Since the intensities of the corresponding Poisson processes *N*^*w*^ and *N*^*d*^, *λ*^*w*^ and *λ*^*d*^, respectively, are given by

they represent the average number of branch consumers who make withdrawals and deposits respectively, in the considered period of time. For our purpose of delimiting the error, the variables (*W*_*i*_)^2^ and (*D*_*i*_)^2^ give us a similar interpretation of what is given by *W*_*i*_ and *D*_*i*_.

Hence, from these formulae, the logical consequence of the model is that the minor is the number of branch users, the minor will be the committed miscalculation. This vaguely seems to indicate that the model makes more accurate predictions for small and medium sized branches.

Note that we use the term *vaguely* to reflect the fact that the notion of branch size seems to be related with the number of branch users, although this has not been specified yet. Actually, there are many equivalent criteria to quantify the size of a branch by bank managers, and one of them is indeed the the number of its clients. In the following sections (section “Comparative statics”) we will return on this point to analyze this with more detail.

## The underlying optimization problem

Since the main target of this paper is to optimize the cash resources of the bank companies across its branches, it is crucial that the quantity that the branch requests from the cash central, *C*_0_, fits perfectly to the branch expenses without surplus. In other words, we propose that *C*_0_ must be small enough to avoid losses to the bankťs company, derived from the fact of holding too much cash in the branch, while the surplus of *C*_0_ should be generating money if it should be deposited in other bank products.

Recall at this point that the remarks made before on *C*_*z*_ can be now applied to *C*_0_: we refer to the fact that we may calculate *C*_0_ either to perfectly fit to the branch expenses without surplus or, at the contrary, we may determine it to exceed these expenses in a “bit more” in order to fulfill the clients demand up to a certain confidence level, even more taking into account that to resolve one problem or the other one has a minuscule difference from the mathematical point of view (equality or inequality constrained minimization).

However, we choose to calculate *C*_0_ for perfectly covering all the branch expenses in order to avoid opportunity costs. And, once we have determined the optimal amount *C*_0_ to minimize costs under the condition of perfectly cover the branch needs, we let to the bank institution the decision of increasing more o less such quantity *C*_0_, depending on their internal rules of functioning.

Taking again the above idea of optimizing the banks objective function with the constrain that *C*_0_ must cover all branch expenses, we arrive in a natural way to the following optimizing problem with constrains:

Let us start with the banks objective function. Remember from section “The mathematical model” that the banks objective function is

As for the constrain of the optimization problem, i.e., *C*_0_ must cover the whole branch expenses, this can be written in mathematical terms as

where *K* includes both expected expenditures and deposits. Note that both terms *E*[*X*_1_] and *E*[*Y*_1_] are known by very simple formulae, as they were deeply analyzed in the former section.

We can not forget the fact that *C*_*z*_ is the upper bound of cash that the bankťs company allows to the branch. Hence, a natural second constrain will be

Note that both constrains together state that

which it is the logical rule to ensure a well functioning of the branch.

Once both the bank objective function and the constrains are specified, the optimization problem turns out to be the following:

To solve this problem, we effect the following change of variables:

Accordingly, the optimization problem turns out to be

We resolve the optimization problem with the first constrain and, later on, we will select from the set of solutions (*x*,*y*) those which verify *x* ≤ 0. For this reason, we solve the minimization program

by substituting *x* = *E*[*X*_1_] − *E*[*Y*_1_] + *K* − *y* into the objective function. If we do so, the solutions to the problem are the roots of the following quartic equation, where, by now, *R* is shorten for *E*[*X*_1_] − *E*[*Y*_1_] + *K* : 

or expanded,

This quartic can be resolved by means of algebraic equations in radicals, following the Galois Theory. This will yield to the desired solution of the optimization cash costs.

For future references, we will denote  to the *optimal policy* for total branch costs. Note that, due to the plain (linear) relationship among variables *C*_0_,*C*_*z*_ and *x*,*y* for the previous change of variables

the optimal return point can be expressed in terms of *x* and *y*, (*x*^∗^,*y*^∗^) or in terms of . We may use both of them indistinctly depending on the context.

As for the optimal value, we will denote this as , and *m*^∗^ = *ε*(*x*^∗^,*y*^∗^). We also use the notation of *m*(*x*,*y*) instead of *ε*(*x*,*y*) when we want to emphasize that our analysis of the objective function is specifically done for finally evaluating it at the optimal return point.

## Comparative statics

Along this paper, some slight approaches on the the accuracy of the model for different branch sizes have been made. The detailed discussion about this will be developed in this section with a comparative statics exercise on the optimal policy *m*^∗^ (or, alternatively, ) in terms of the primitive parameters *R* and *μ*.

### The branch size

Let us start this paragraph by making some remarks about the concept of **branch size**.

Intuitively, the notion of branch size is identified with the volume of its turnover. Actually, there are many criteria to quantify the size of a branch: the volume of its credits, the number of its clients, the number of its staff or the volume of its deposits, among others. *In practice* (i.e., among bank managers), the most accepted criterion to measure the size of a branch is simply to quantify its *total needs of cash* and then, to state that branch size as an increasing function of these needs. In short, bank managers assume that the bigger branch sizes correspond to the mayor needs of cash of the branch, related to mayor moves of money (entries and exits).

Even though we used *R* before in order to shorten the notation (of the quartic equation), we do make now a formal definition: *R* is defined as

and it represents the *total needs of cash of the branch*. For the reasons exposed before, we will assume from now on that the parameter *R* quantifies specifically the size of the branch. As a parameter, *R* takes values on [0,+*∞*), although for simplicity, in practice *R* is considered as belonging to one of the three categories: small, medium and big size,

Then, regarded simply as the branch size, the *parameter* *R* will allow us to calibrate for which sizes our model is more or less accurate. Recall that *R* is the second indicator we define since actually we introduced at the end of section “The mathematical model” the parameter *μ* as an index for measuring the cash flow fluctuations of the branch.

As we mentioned before, the main objective of this section is to do some comparative static exercises on the optimal policy *m*^∗^ (or, alternatively, ) in terms of the primitive parameters *R* and *μ*. Let us start with *R*, by finding the sign of  to state in which cases *m* is increasing or decreasing on *R*.

Let us observe first that *m* is function of *y*, *m* = *m*(*y*), via the objective function

with the change *x* = *R* − *y*, that is,

so we may calculate . This partial derivative is always positive since this is the result of algebraic operations (sum and product) among positive quantities. Specifically,

which is positive as all terms involved so are as we claimed. Actually, since the second addend is positive with no doubt, we focus on the first addend: while it is clear that the denominator of the first term is positive, note that also it is the numerator of the fraction since *y*(=*C*_*z*_) ≥ *R* for well functioning of the branches, so

This shows that the optimal value *m*^∗^ is increasing on the optimal policy *y*^∗^.

Let us note that this fact, *m*^∗^ is increasing on *y*^∗^, is not related with the parameters of the model but it is simply a logical consequence of the model: mayor will be this upper bound (maximum of cash) , higher will be the optimal value for costs. This statement *should* be kept in mind for the bank companies when deciding (or not) to add the “bit more” over  for precautionary motives according with their internal rules of functioning, independently either of the size of the branches or the fluctuations of their cash balance.

On the other hand, the function *m*() may also be regarded as a function of *R*:

Henceforth we may calculate :

which could be positive or negative, depending on the values of both  and .

We note that we have the following chain of logical implications:

since

because the subtrahend → *∞*. A similar argument allows us to state that

On the other hand, it is nor difficult to see that *x*^2^*y* is increasing on *y* since

that is, if *y*↗ ⇒*x*^2^*y*↗. Thus, combining both results, we have that

1. when .
2. When .
  In other words, since *y* = *C*_*z*_, the above result states that
3. When *C*_*z*_ → 0, then *m*^∗^ is decreasing on *R*.
4. When *C*_*z*_→*∞* then *m*^∗^ is increasing on *R*.

Along this paper we have rejected for simplicity some features of *C*_*z*_ to center our attention on the main requisite on *C*_*z*_ of minimizing costs to the bankťs company. At this point, we return to the fact that *C*_*z*_ (maximum of cash) is fixed by the bankťs company for each branch, attending some parameters, particularly, the size of the branch.

Although other considerations (like geographic location) may influence, in the reality *C*_*z*_ is fixed by the bank company in a directly proportional way to the branch size: higher is the branch size, bigger will be *C*_*z*_. That is, if

This result states that in the category of small branches (branches for which size *R* takes small values) the optimal value for branch costs *m*^∗^ decreases in despite of *R* increases. This may be interpreted in the sense that the bank company can expected to have the same optimal value for costs *m*^∗^*for all branches of this category, even for branches with sizes in a neighborhood of small size*. That is, for small/medium branches.

However, the opposite phenomena occurs for the category of big branches: dealing with them, the bank company may expect that the optimal costs will blow up as the size of the branches increase.

Now we return to the central optimization problem of this paper, which yields us to discuss the features of the Lagrange multiplier associated to this program. This allows us to state some complementary properties of the index *R*.

While we have solved the minimization program

by substituting *x* = *E*[*X*_1_] − *E*[*Y*_1_] + *K* − *y* into the objective function, one also would have resolved this by applying the Lagrange method: let us see then which features the associated Lagrange multiplier exhibits and which economic implications could be derived from this.

When the objective is to minimize the cost function subject to the “output constraint”, it is well known that the Lagrange multiplier turns out to be the marginal cost of “production” that is, the increase in total costs when one more unit of output is permitted. Note that the “output” in our constrain corresponds to the branch needs of cash (or alternatively, the branch size), *R*.

By incorporating *R*, the minimization problem written in terms of *x* and *y* as variables (as before) turns out to be

As it is well known, the solution of this constrained optimization problem can be found as well by using the so-called Lagrangian method. If we define the Lagrangian as , the equations given by this Lagrange multiplier method are

where the multiplier *λ* has an economic interpretation. As it is well known,

represents the rate of change of the optimal return point  when *R* increases one unit.

From the first and second equations, we have that

From algebraic computation, it is clear that *λ* is increasing function of both *x*^2^*y* and *xy*^2^: actually, if *x*^2^*y* or *xy*^2^ increase, then  and  decrease, so  and  increase as well, even more if both are multiplied by non negative constants. Finally, to determine that both *x*^2^*y* and *xy*^2^ are increasing functions of *R*, we simply use the test of first derivative: since *x* + *y* = *R* then

Hence,

As a result of this, we have the following sequence of implications:

as  and  and *m*^∗^ are linearly related. As a consequence of this,

, and
,

The economical interpretation of this result is the following: this outcome states that, as the branch size increases, the rate of changes in the optimum for branch costs increases as well. This means that the situation of branch costs *deteriorates* at major speed when dealing with branches of big size. All the contrary, the situation of possible changes on optimal costs for the branch of small size diminishes (improves) for small values of *R*.

### The fluctuations of the cash flow

This paragraph is devoted to analyzing how the parameter *μ*, defined in section “The mathematical model”, influences the behavior of the optimal return point *m*^∗^. Let us start then with some remarks about the meaning of the parameter *μ*.

Recall that *μ* appears at the first addend of the objective function

where *A* = *μ*^2^*t* is the variance of daily changes in the cash balance and *μ* stated for the amount of euros that the branch cash balance increases or decreases in some small fraction of a working day . Then, as we established in section “The mathematical model”, *μ* shows the branch users behavior as it indicates how active the branch users are in form of *fluctuations* of the branch cash balance.

Recall also that, in practice, *μ* is strongly directly related with the *geographic location* of the branch since the fluctuations of the cash balance -in the major part of the cases- are caused by some *physical reason* (in a similar way that a physical phenomena disturbs the state of rest of any object, changing this initial *state of rest* -with a graphical representation close to a straight line- into a *motion state*-represented by a periodic wave motion, bigger or smaller depending on the intensity of the disturbs).

In our particular context, this physical phenomena which disturbs the branch cash flow may be identified with the proximity to a *cash center*, in the wide sense of this expression: that is, understanding that a cash center is an industrial, financial, shopping center or whatever other physical zone where the moves of money are higher than usual.

Thus, for our purposes of calibrating the model, *μ* will quantify the fluctuations in the branch cash flow and *μ* ∈ [0,+*∞*). However, due to the direct relationship exposed before between the fluctuations of the cash flow and the physical reason that causes the fluctuations (cash center), *μ* could be interpreted as geographic location of the branch -in reference to a cash center- as well.

As function of *x*^∗^,*y*^∗^, *m*^∗^ is

In a similar way, *m*^∗^ may be regarded as a function of *μ*:

From this expression it can be derived that

since the factor  is negative as *x*^∗^ < 0 while the rest of factor (*γ*,*t*,*y*^∗^) are positive. As a consequence of this, *m*^∗^ is decreasing on *μ* in all cases.

In contrast with the results based on the parameter *R*, where the result clearly depends on the values of *R*, the case of the parameter *μ* can be written in absolute terms: *for all the values ofμ* the optimal value for branch costs *m*^∗^ is always decreasing on *μ*. That may be interpreted as, if the fluctuations in the branch cash balance increases, the optimum for branch costs decreases: specifically, the situation of branch costs *improves* when dealing with branches of big fluctuations in the cash flow, that is, when dealing with branches closely located near to cash centers.

## Conclusions

This paper offers a mathematical model which reflects specific and relevant features of bank branch cash management with important implications for bank efficiency. Many banking systems are going through intense processes of consolidation which include not only mergers and acquisitions but also efficiency plans. In these plans, the number of bank branches, their size and different aspects of their management are a critical feature for determining the success of the restructuring process in terms of efficiency gains.

The model in this paper focuses on optimization of cash inventories which has been always a critical feature of financial intermediation given the maturity transformation that banks face by converting short-term liquidity to long-term funding.

Our model provides some simple but easy to implement and effective tools to improve bank branch optimization of cash inventories. It relies on a series of simplifying assumptions that, however, do not affect the practical implementation of the model.

The model and the results themselves provide contributions to the extant literature in, at least, three dimensions. First of all, the model offers a way of managing and monitoring cash optimization that reduces the average branch management costs to a significant extent. Secondly, the magnitude of the efficiency gains of applying such cash management model are mainly dependent on the size of the branch. In particular, optimizing cash inventories seems to be more feasible at relatively small or medium-sized branches. However, in the case of large branches, there seems to be little room for efficiency improvements, at least in what cash efficiency issues are concerned. Third, the model also shows that branch cash management costs can be reduced to a large extent in those branches where both the outflows and inflows of cash are particularly intense (high fluctuations in the cash balance), such as the case of branches located to particularly active urban areas or cash centers.

Our model also has implications for bank restructuring processes in what it provides a way to evaluate efficiency improvements from changing branch size (or simply closing branches) from the cash management perspective. The model as well suggests that the (significant) differences in the size of bank branches across banking sectors internationally may respond, inter alia, to different ways of dealing with branch cash management.

Finally, it is important to point out that the simplicity of the mathematical model proposed makes it particularly feasible to implement. It is also worth noting that some of the simplifying assumptions of the model (such as making the maximum of cash available equal for each branches irrespective of their location) do not affect the overall validity of the results as banks tend to apply their branch cash management methods in a very standard way for all the branches within a bank.

## Abbreviations

i.i.i.: independent and identically distributed
IT: Information technology
SLP: Stochastic linear program
